# Supplementary material for: Joint, multifaceted genomic analysis enables diagnosis of diverse, ultra-rare monogenic presentations
Source: Nat Commun. 2025 Aug 7;16:7267. doi: 10.1038/s41467-025-61712-2 (PMC12328722; doi:10.1038/s41467-025-61712-2)
Supplement: Supplementary file 2 — Description of Additional Supplementary Files [file 41467_2025_61712_MOESM2_ESM.pdf]

Description of Additional Supplementary Files for

Joint, multifaceted genomic analysis enables diagnosis of diverse, ultra-rare monogenic presentations

Shilpa Nadimpalli Kobren[1,*], Mikhail A. Moldovan[1,*], Rebecca Reimers[2,3], Daniel Traviglia[1], Xinyun Li[4,5], Danielle Barnum[6,7], Alexander Veit[1], Rosario I. Corona[8], George de V. Carvalho Neto[8], Julian Willett[9], Michele Berselli[1], William Ronchetti[1], Stanley F. Nelson[8], Julian A. Martinez-Agosto[8], Richard Sherwood[7], Joel Krier[10], Isaac S. Kohane[1], Undiagnosed Diseases Network, Shamil R. Sunyaev[1,†]

*Indicates equal contribution
[†]Email: shamil_sunyaev@hms.harvard.edu

1 Department of Biomedical Informatics, Harvard Medical School, Boston, MA
2 Scripps Research Translational Institute, La Jolla, CA
3 Division of Pediatric Genetics, University of California, San Diego, CA
4 Department of Biostatistics, Harvard T.H. Chan School of Public Health, Boston, MA
5 Interdepartmental Neuroscience Program, Yale University, New Haven, CT
6 Vrije Universiteit Amsterdam, Medical School of V, De Boelelaan 1105, 1081 HV, Amsterdam, Netherlands
7 Division of Genetics, Department of Medicine, Brigham and Women's Hospital and Harvard Medical School, Boston, MA
8 Department of Human Genetics, David Geffen School of Medicine, University of California, Los Angeles, CA
9 Department of Pathology and Laboratory Medicine, NewYork-Presbyterian Weill Cornell Medical Center, New York, NY
10 Department of Genetics, Atrius Health, Boston, MA

Supplementary Data Tables

Each table can be found in its own sheet (tab) in the Excel spreadsheet.

Supplementary Data 1. Candidate vs. Decoy Genes and Final Clinical Scores
Clinical team applying protocol to assign final clinical scores (descending values from most to least compelling) was blinded to gene labels (i.e., decoy or candidate) and *in silico* variant pathogenicity predictions. Columns (from left to right) correspond to: gene name (HGNC ID), brief free-text clinical description of patient, final clinical score, and gene label.

Supplementary Data 2. *De novo* SNV and indel variants in protein-coding genes in UDN patients

We identified putative *de novo* variants across every sequenced trio as those with an alternate allele frequency > 0.2 in the proband, zero supporting reads in either parent, and an annotated Ensembl (v108) gene ID with "protein coding" biotype (including "upstream_gene_variant" and "downstream_gene_variant" types). NovoCaller was used to assign a posterior probability to each putative *de novo* based on aligned reads in the trio and unrelated individuals. All *de novo* variants with a posterior probability > 0.7 from 872 affected individuals are listed here. Columns (from left to right) correspond to:

1. transformed patient identifiers
2. chromosome (Ensembl format) from GRCh38
3. 1-indexed position
4. reference allele
5. alternate allele
6. semi-colon-separated read depth in kid, mom and dad. Format is: # reads supporting reference allele, # reads supporting alternate read / total aligned reads
7. NovoCaller posterior probability (ranges from 0 to 1)

Supplementary Data 3. *De novo* recurrence findings using AI pathogenicity predictors for missense variants

We computed our *de novo* recurrence statistic (RaMeDiES-DN) across confident *de novo* variants from 872 affected individuals as described in Methods and listed in Supplementary Data 2. We applied two AI-based pathogenicity predictors, AlphaMissense and PrimateAI-3D, which score missense variants only. Columns (from left to right) correspond to:

1. gene name (HGNC ID),
2. Ensembl gene ID,
3. Cauchy-combined *P*-value (uncorrected for multiple hypotheses),
4. patient UDN diagnosis status (correct gene match, UDN clinician-derived candidate, *incorrect* gene match with an alternate diagnosis listed, or blank),
5. unadjusted patient–gene HPO term similarity score (computed with Phrank),
6. whether the gene is a known developmental disorder gene[1]
7. patient sex,
8. variant consequence,
9. variant functionality score(s),
10. a comment column reminding users that a patient–gene similarity score may be 0 despite a correct diagnosis for the patient if HPO annotations for the patient or gene were limited,

11. the GeneBayes constraint-weighted Q-value (uncorrected for multiple hypotheses),
12. whether the gene's P-value passed the Bonferroni significance threshold,
13. whether the gene's Q-value passed the equivalent threshold,
14. whether the gene's Q-value is within FDR 5%, and
15. whether the gene's Q-value is within FDR 10%.

Supplementary Data 4. *De novo* recurrence findings using four pathogenicity predictors for exonic variants

We computed our *de novo* recurrence statistic (RaMeDiES-DN) across confident *de novo* variants from 872 affected individuals as described in Methods and listed in Supplementary Data 2. We applied four pathogenicity predictors, three of which score missense variants only (i.e., AlphaMissense, PrimateAI-3D, and REVEL) and one of which additionally scores nonsense and indel variants (CADD). Columns (from left to right) correspond to:

1. gene name (HGNC ID),
2. Ensembl gene ID,
3. Cauchy-combined P-value (uncorrected for multiple hypotheses),
4. patient UDN diagnosis status (correct gene match, UDN clinician-derived candidate, *incorrect* gene match with an alternate diagnosis listed, or blank),
5. unadjusted patient–gene HPO term similarity score (computed with Phrank),
6. whether the gene is a known developmental disorder gene[1]
7. patient sex,
8. variant consequence,
9. variant functionality score(s),
10. a comment column reminding users that a patient–gene similarity score may be 0 despite a correct diagnosis for the patient if HPO annotations for the patient or gene were limited,
11. the GeneBayes constraint-weighted Q-value (uncorrected for multiple hypotheses),
12. whether the gene's P-value passed the Bonferroni significance threshold,
13. whether the gene's Q-value passed the equivalent threshold,
14. whether the gene's Q-value is within FDR 5%, and
15. whether the gene's Q-value is within FDR 10%.

Supplementary Data 5. RaMeDiES-DN and DeNovoWEST ranked genes on DDD+GeneDx+RUMC cohort

We ran both DeNovoWEST and a CADD-only version of RaMeDiES-DN on autosomal, lifted-over exonic *de novo* variants from the combined DDD (7,310 probands), GeneDx (13,903 probands), and RUMC (1,683 probands) cohort published in *Nature*, 2020.[2] Note that *de novos* that were on chromosome X, were not in standard VCF format (i.e., non-normalized or left-aligned indels), or could not be lifted over to GRCh38 were

excluded, and only probands with 1+ remaining *de novo* variants were retained for analysis. Columns (from left to right) correspond to:

1. gene name (HGNC ID),
2. Ensembl gene ID,
3. Original gene categorization from N*ature* 2020, where "consensus" refers to disease genes that appeared on diagnostic gene panels from all three centers: DDD, GeneDx, and RUMC, "discordant" refers to disease genes that appeared on a diagnostic gene panel in only one or two of the three centers, "novel" refers to disease genes that did not appear on any diagnostic gene panels in 2020 but were identified as significant by DeNovoWEST and published in N*ature* 2020, and "." refers to any other gene.
4. Gene is associated with an autosomal dominant disease as reported in OMIM (TRUE) or otherwise (.)
5. A knockout of this gene in mouse was viable, subviable, lethal, or unknown (.) as reported by the International Mouse Phenotyping Consortium (IMPC)
6. RaMeDiES-DN uncorrected P-value
7. Pipe-delimited list of *de novo* variants in gene (chromosome, position, reference allele, alternate allele, and patient ID)
8. DeNovoWEST uncorrected P-value
9. GeneBayes-weighted RaMeDiES-DN Q-value

Supplementary Data 6. RaMeDiES-DN meta-analysis versus full cohort analysis on DDD+GeneDx+RUMC

We ran two versions of RaMeDiES-DN and compared the results. The first run was on patient-specific *de novo* variants from DDD+GeneDx+RUMC. The second run was on the DDD, GeneDx, and RUMC patient cohorts in turn followed by a meta-analysis RaMeDiES-DN run to combine deidentified, summary-level mutational target statistics from each of the three cohorts. Columns (from left to right) correspond to:

1. gene name (HGNC ID),
2. Ensembl gene ID,
3. RaMeDiES-DN meta-analysis uncorrected p-value
4. GeneBayes-weighted RaMeDiES-DN meta-analysis Q-value
5. RaMeDiES-DN uncorrected P-value on DDD patients alone
6. RaMeDiES-DN uncorrected P-value on GeneDx patients alone
7. RaMeDiES-DN uncorrected P-value on RUMC patients alone
8. RaMeDiES-DN uncorrected P-value on DDD+GeneDx+RUMC patients together
9. GeneBayes-weighted RaMeDiES-DN Q-value on DDD+GeneDx+RUMC patients together

Supplementary Data 7. Individual-level compound heterozygous findings
We computed our individual-level compound heterozygous statistic across rare inherited variants from 854 affected individuals as described in Methods. Columns (from left to right) correspond to:

1. Cauchy-combined P-value (uncorrected for multiple hypotheses),
2. gene name (HGNC ID),
3. Ensembl gene ID,
4. patient UDN diagnosis status (correct gene match, UDN clinician-derived candidate, *incorrect* gene match with an alternate diagnosis listed, or blank),
5. unadjusted patient–gene HPO term similarity score (computed with Phrank),
6. patient sex,
7. maternal variant consequence (if known),
8. maternal variant functionality scores (if known),
9. paternal variant consequence (if known),
10. paternal variant functionality scores (if known) .

Supplementary Data 8. Candidate genes per patient cluster
Patients were clustered into phenotypically-similar subgroups, and then compelling genes were selected per patient in each cluster as described in Methods. The set of genes per patient cluster was used as a query for gene set enrichment analysis. Columns (from left to right) correspond to: pathway ID ("HC" indicates hierarchical clustering), gene name (HGNC ID), gene type (i.e., "denovo" indicates a compelling *de novo* variant was present, "comphet" indicates a compelling compound heterozygous variant was present, and "augmented" indicates a known diagnosis that was established through the UDN process), patient diagnosis status, and unadjusted patient–gene HPO term similarity score (computed with Phrank). Note that some correct diagnoses may have a patient–gene phenotype similarity score of zero if either the patient or the gene did not have any annotated HPO terms.

Supplementary Data 9. All enriched biological pathways across patient clusters
We performed gene set enrichment analysis using Reactome and KEGG biological pathways as described in Methods. The query genes selected per patient cluster include known diagnoses achieved through UDN analyses as well as computationally-derived *de novo* and compound heterozygous candidates, as described in Methods and listed in Supplementary Data 8. Columns (from left to right) correspond to: pathway ID (asterisk indicates a patient–gene pair that also appears earlier in the list), pathway name, pathway size, GSEA *p*-value adjusted for multiple testing using g:Profiler's Statistical Correction Scheme, cluster ID and effective gene set query size, gene name (HGNC ID), Ensembl gene ID, patient diagnosis status, unadjusted patient–gene HPO term similarity score (computed with Phrank), patient sex, variant consequence (if known), variant functionality scores (if

known). Note that the effective gene set query size can be different for the same cluster ID between Reactome and KEGG enriched pathways. The original gene set query for each patient cluster is intersected with the set of annotated genes across all Reactome or all KEGG pathways respectively; some genes may only be present in one of the two data sources.

References

1. Kingdom, R., Beaumont, R. N., Wood, A. R., Weedon, M. N. & Wright, C. F. Genetic modifiers of rare variants in monogenic developmental disorder loci. *Nat. Genet.* **56**, 861–868 (2024).
2. Kaplanis, J. *et al.* Evidence for 28 genetic disorders discovered by combining healthcare and research data. *Nature* **586**, 757 (2020).
