# Supplementary material for: Joint, multifaceted genomic analysis enables diagnosis of diverse, ultra-rare monogenic presentations
Source: Nat Commun. 2025 Aug 7;16:7267. doi: 10.1038/s41467-025-61712-2 (PMC12328722; doi:10.1038/s41467-025-61712-2)
Supplement: Supplementary file 4 — Reporting Summary [file 41467_2025_61712_MOESM4_ESM.pdf]

nature portfolio

Corresponding author(s): Shamil R. Sunyaev

Last updated by author(s): Feb 21, 2024

Reporting Summary

Nature Portfolio wishes to improve the reproducibility of the work that we publish. This form provides structure for consistency and transparency in reporting. For further information on Nature Portfolio policies, see our Editorial Policies and the Editorial Policy Checklist.

Statistics

For all statistical analyses, confirm that the following items are present in the figure legend, table legend, main text, or Methods section.

n/a	Confirmed	
☐	☒	The exact sample size (n) for each experimental group/condition, given as a discrete number and unit of measurement
☐	☒	A statement on whether measurements were taken from distinct samples or whether the same sample was measured repeatedly
☐	☒	The statistical test(s) used AND whether they are one- or two-sided<br>*Only common tests should be described solely by name; describe more complex techniques in the Methods section.*
☒	☐	A description of all covariates tested
☐	☒	A description of any assumptions or corrections, such as tests of normality and adjustment for multiple comparisons
☐	☒	A full description of the statistical parameters including central tendency (e.g. means) or other basic estimates (e.g. regression coefficient) AND variation (e.g. standard deviation) or associated estimates of uncertainty (e.g. confidence intervals)
☐	☒	For null hypothesis testing, the test statistic (e.g. F, t, r) with confidence intervals, effect sizes, degrees of freedom and P value noted<br>*Give P values as exact values whenever suitable.*
☒	☐	For Bayesian analysis, information on the choice of priors and Markov chain Monte Carlo settings
☐	☒	For hierarchical and complex designs, identification of the appropriate level for tests and full reporting of outcomes
☐	☒	Estimates of effect sizes (e.g. Cohen's d, Pearson's r), indicating how they were calculated

Our web collection on statistics for biologists contains articles on many of the points above.

Software and code

Policy information about availability of computer code

Data collection	Data was retrieved from the Undiagnosed Diseases Network (UDN) Data Management and Coordinating Center programmatically using their Metrics and Reports download.
Data analysis	CGAP pipeline (upstream Sentieon, SNV germline) version 29cefcec. Sentieon joint calling version 202112.02. Bcftools and htslib version 1.14. FASTQC version 08-01-19. Somalier version v0.2.15. KING version 2.3.0, Ensembl VEP version 108, slivar v0.2.7, novoCaller (CGAP, 2022), DeNovoWEST v1.0.0, Phrank v2018-12-13, R cluster package v2.1.4, R tm package v0.7.11, R quanteda package v3.3.1, gProfiler version e108_eg55_p17, RaMeDiES custom software implementation (v1.0.0) available on GitHub: https://github.com/hms-dbmi/RaMeDiES

For manuscripts utilizing custom algorithms or software that are central to the research but not yet described in published literature, software must be made available to editors and reviewers. We strongly encourage code deposition in a community repository (e.g. GitHub). See the Nature Portfolio guidelines for submitting code & software for further information.

Data

Policy information about availability of data

All manuscripts must include a data availability statement. This statement should provide the following information, where applicable:

- Accession codes, unique identifiers, or web links for publicly available datasets
- A description of any restrictions on data availability
- For clinical datasets or third party data, please ensure that the statement adheres to our policy

Data was collected centrally by the UDN Data Management and Coordinating Center (independently of this work) as described in the Manual of Operations (https://undiagnosed.hms.harvard.edu/research/udn-manual-of-operations/). The deidentified genome data, transcriptome data, and corresponding phenotype data in the form of HPO terms used in this study have been deposited in the dbGaP database under accession phs001232.v5.p2 [https://www.ncbi.nlm.nih.gov/projects/gap/cgi-bin/study.cgi?study_id=phs001232.v5.p2]. Genome-wide, rare SNV and indel variants and HPO codes for UDN participants included in this study are queryable in our public-facing browser [https://dbmi-bgm.github.io/udn-browser/]. Standardized phenotype data and candidate genes and variants used in this study have been submitted to the Matchmaker Exchange database [https://www.matchmakerexchange.org/]. Variant-level data, clinical significance and supporting evidence, demographic information, and phenotype information for all candidate and diagnostic variants, including those identified through this study, have been submitted to the ClinVar database [https://www.ncbi.nlm.nih.gov/clinvar/]. Identifiable patient data is available only to UDN investigators under restricted access to protect patient privacy in compliance with patient consent. Other relevant, deidentified patient-specific clinical information available to researchers involved in this study but not available publicly may be shared on a case-by-case basis at the discretion of the corresponding clinical team if it is directly related to diagnosing or potentially treating the patient. Queries can be directed to udncc@hms.harvard.edu. Plasmids used in the MPSA experiment have been deposited to Addgene under accession numbers 240805 ("plentiMPSA SAentry PuroR") and 240806 ("plentiMPSA SDentry PuroR") [https://www.addgene.org/].

Research involving human participants, their data, or biological material

Policy information about studies with human participants or human data. See also policy information about sex, gender (identity/presentation), and sexual orientation and race, ethnicity and racism.

Reporting on sex and gender	Sex was determined directly from whole genome sequencing for UDN participants included in this study. The distribution of males and females included in the dataset can be found in Figure 1e. Only genes on autosomes (chromosomes 1-22) were included in our joint analysis, and therefore there are no sex-based analyses.
Reporting on race, ethnicity, or other socially relevant groupings	The ancestry of UDN participants included in this study is depicted as a PCA plot in Figure 1b. Individuals with detectable levels of consanguinity were excluded for reasons outlined in our Methods section. Our recessive inheritance models utilize the numbers of inherited rare variants directly observed in each trio, and so population structure or stratification was not used nor is it relevant for our analyses.
Population characteristics	The age at first symptom onset for UDN participants is shown in Figure 1d; this ranges from birth through adulthood. The clinician-reported primary symptom category for all UDN participants is shown in Figure 1c, but this is not used as a covariate in our study (all participants are considered together). All participants are undiagnosed at the time of enrollment. Information about current medications and treatments is collected at the time of enrollment and evaluation, but this is not used as a metric for inclusion.
Recruitment	Recruitment strategies are detailed in section 6.4 of the IRB. Briefly, recruitment can happen via a variety of sources, including the UDN website, healthcare providers, social media, informational flyers, emails, publicity, or analysis of deidentified claims data. Inclusion and exclusion criteria for applicants are detailed in sections 6.2.1 (inclusion) and 6.2.2 (exclusion). Briefly, applicants must be undiagnosed (i.e. not have a diagnosis suggested on record review or explaining all objective findings for which an application was submitted), and must be willing to share data. These recruitment strategies may be biased in that patients who are ultimately accepted likely have had the means to see 2+ specialist physicians who were able to put together a complete and detailed application package, and patients must be willing to share identifiable data and biomaterials.
Ethics oversight	Central IRB with the NIH #15HG0130

Note that full information on the approval of the study protocol must also be provided in the manuscript.

Field-specific reporting

Please select the one below that is the best fit for your research. If you are not sure, read the appropriate sections before making your selection.

☒ Life sciences ☐ Behavioural & social sciences ☐ Ecological, evolutionary & environmental sciences

For a reference copy of the document with all sections, see nature.com/documents/nr-reporting-summary-flat.pdf

Life sciences study design

All studies must disclose on these points even when the disclosure is negative.

Sample size	No sample size calculation was performed; all participants currently enrolled in the Undiagnosed Diseases Network were considered. Our analysis (i.e., genes that harbored de novo variants across multiple affected, unrelated individuals) shows that the size of this dataset was

	sufficient for some statistically-significant findings, but part of our work shows that a similar analysis on a larger cohort of patients may reveal new findings.
Data exclusions	Only participants with trio whole genome sequencing and a suspected sporadic/recessive inheritance (i.e., affected child, two unaffected parents) were initially considered in our de novo and compound heterozygous analyses. Any trios where expected relatedness coefficients were violated were excluded (including close relationship between parents or lack of relationship between parent-child, as described in methods). Individuals with suspected mosiacism were excluded. Only one affected individual per family was included.
Replication	Findings from our massively parallel splicing assay (MPSA), specifically the validation rate of specific variants predicted to alter splicing, were successfully reproduced across six biological replicates as described in Methods.
Randomization	This is not relevant to our study, because participants were not allocated into experimental groups: all individuals were considered together for a cohort analysis, regardless of their diagnosis status, primary symptom category, ancestry, or otherwise.
Blinding	Blinding was not relevant for our study, because participants were never allocated into different groups. We performed a cohort-level analysis and considered all UDN patients (diagnosed or undiagnosed, across all clinical centers and without separating by primary phenotype) in order to see if any genes were recurrently implicated across patients.

Reporting for specific materials, systems and methods

We require information from authors about some types of materials, experimental systems and methods used in many studies. Here, indicate whether each material, system or method listed is relevant to your study. If you are not sure if a list item applies to your research, read the appropriate section before selecting a response.

Materials & experimental systems

n/a	Involved in the study
☒	☐ Antibodies
☐	☒ Eukaryotic cell lines
☒	☐ Palaeontology and archaeology
☒	☐ Animals and other organisms
☒	☐ Clinical data
☒	☐ Dual use research of concern
☒	☐ Plants

Methods

n/a	Involved in the study
☒	☐ ChIP-seq
☒	☐ Flow cytometry
☒	☐ MRI-based neuroimaging

Eukaryotic cell lines

Policy information about cell lines and Sex and Gender in Research

Cell line source(s)	American Type Culture Collection (ATCC), cell lines HepG2 (catalog #HB-8065, liver, isolated from a white, male, 15-year-old) and SK-N-SH (catalog #HTB-11, neural-like, isolated from a female 4-year-old)
Authentication	We compared RNA-Seq and genome sequencing data to reference samples to authenticate cell lines.
Mycoplasma contamination	Cells were routinely tested for mycoplasma contamination via qPCR; cells consistently tested negative.
Commonly misidentified lines (See ICLAC register)	None used.

Plants

Seed stocks	*Report on the source of all seed stocks or other plant material used. If applicable, state the seed stock centre and catalogue number. If plant specimens were collected from the field, describe the collection location, date and sampling procedures.*
Novel plant genotypes	*Describe the methods by which all novel plant genotypes were produced. This includes those generated by transgenic approaches, gene editing, chemical/radiation-based mutagenesis and hybridization. For transgenic lines, describe the transformation method, the number of independent lines analyzed and the generation upon which experiments were performed. For gene-edited lines, describe the editor used, the endogenous sequence targeted for editing, the targeting guide RNA sequence (if applicable) and how the editor was applied.*
Authentication	*Describe any authentication procedures for each seed stock used or novel genotype generated. Describe any experiments used to assess the effect of a mutation and, where applicable, how potential secondary effects (e.g. second site T-DNA insertions, mosiacism, off-target gene editing) were examined.*
